# Psychological Well-Being as an Independent Predictor of Exercise Capacity in Cardiac Rehabilitation Patients With Obesity

**DOI:** 10.3389/fpsyg.2019.02973

**Published:** 2020-01-28

**Authors:** Giada Pietrabissa, Gianluca Castelnuovo, Gian Mauro Manzoni, Roberto Cattivelli, Enrico Molinari, Luca Alessandro Gondoni

**Affiliations:** ^1^Istituto Auxologico Italiano IRCCS, Psychology Research Laboratory, San Giuseppe Hospital, Verbania, Italy; ^2^Department of Psychology, Catholic University of the Sacred Heart, Milan, Italy; ^3^Faculty of Psychology, eCampus University, Como, Italy; ^4^Istituto Auxologico Italiano IRCCS, Cardiac Rehabilitation Unit, San Giuseppe Hospital, Verbania, Italy

**Keywords:** physical activity, exercise capacity, psychological well-being, cardiac rehabilitation, obesity

## Abstract

**Objective:** Exercise capacity (EC) is a well-established predictor of cardiovascular health. It is notoriously influenced by several factors, but the independent effect of psychological well-being (PWB) on EC has not yet been explored. The present study aims to investigate (1) whether PWB is an independent predictor of EC over and above selected demographic, behavioral, and biomedical parameters in a sample of CR patients with obesity and (2) whether PWB is a stronger predictor of EC than the other variables.

**Methods:** Data from 1968 patients were collected at the time of their inclusion in a cardiac rehabilitation (CR) program and retrospectively analyzed in a cross-sectional study. Since cardiorespiratory parameters defined in normal weight populations differ from those of their obese counterparts, an *ad hoc* validated formula taking body mass index (BMI) into consideration was used to predict EC.

**Results:** A multiple regression analysis revealed left ventricular eject fraction (LVEF) to be the strongest predictor of EC, followed by PWB, type 2 diabetes (DM), smoking status, atrial fibrillation (AF), and education. Bayesian evaluation of informative hypotheses corroborated LVEF as the best predictor of EC, and confirmed the superiority of PWB over and above DM and smoking status in influencing EC.

**Conclusion:** These findings strengthen the link between psychological and physical health, suggesting a better PWB is associated with greater EC. Prompt screening of a patient’s mood and readiness to perform an active lifestyle would therefore enhance the long-term health benefits of CR.

## Introduction

Cardiovascular disease (CVD) is a leading cause of disability and premature death, globally, and substantially contributes to the escalating costs of healthcare (Roth et al., 2017). The American Heart Association (AHA) has established a sedentary lifestyle as a major modifiable risk factor for CVD (Thompson et al., 2003). However, despite overwhelming evidence promoting an active lifestyle in both primary prevention and formal cardiac rehabilitation (CR), evidence suggests that patients with CVD often fail to maintain physical activity (PA) recommendations (Dontje et al., 2014; Yates et al., 2017).

Still, a low level of PA increases the risk of developing other chronic conditions including diabetes, hypertension, obesity (Wing and Hill, 2001), and depression (Pollock, 2001; Dibben et al., 2018). Findings from a meta-analysis of 63 randomized studies evaluating the efficacy of different CR programs–with and without exercise–in patients with documented ischemic heart disease (IHD) showed that exercise alone produced a significant reduction in diverse causes of mortality (Clark et al., 2005). Thus, exercise capacity (EC) is a valuable measure for the control and treatment of CVD, and its assessment provides important information to guide exercise prescription (Demers et al., 2001; Banerjee et al., 2012). Assessment includes subjective evaluation of an individual’s exercise tolerance and objective exercise test (ET) results.

Besides the well-known effect of age and gender, which continue to be the basis of the equation most frequently used to calculate exercise intensity (Ahmadian et al., 2013), evidence exists for the influence of body mass index (BMI, kg/m^2^) on EC (Gondoni et al., 2010; Gong et al., 2013). Obesity is constantly increasing in prevalence (Pietrabissa et al., 2012; Holmgren et al., 2017) and the cardiovascular benefits obtained from increasing PA are greater than those from dietary control to lose weight.

Sufficient information is also found in the medical literature on the role of atrial fibrillation (AF) (Osbak et al., 2012; Zakeri et al., 2014), left ventricular ejection fraction (LVEF) (Wong and Yeo, 2010), type 2 diabetes (DM) (Awotidebe et al., 2014), smoking status (Mesquita et al., 2015), and educational level (Witham et al., 2006) in predicting aerobic capacity in patients with CVD.

Although evidence exists for the negative impact of impaired EC on perceived psychological well-being (PWB) in the cardiac population (Wang, 2018), the role of psychosocial domains on EC remains unclear (Chiala et al., 2018). Also, to our knowledge, no study has yet tested the independent effect of PWB in determining the maximum amount of physical exertion that a patient with both CVD and obesity can sustain. Understanding this effect could assist in the design of tailored assessments and intervention procedures in CR.

The present cross-sectional study aims to investigate (1) whether PWB is an independent predictor of EC over and above a series of selected demographic, behavioral, and biomedical parameters in a sample of patients with CVD and obesity, and (2) whether PWB is a stronger predictor of EC than the other selected variables.

## Materials and Methods

### Participants

From January 2012 to June 2019, relevant data were collected from 1968 consecutive patients (1348 males) with CVD and obesity who were referred to a single clinical center (Istituto Auxologico Italiano IRCCS, San Giuseppe Hospital, Verbania, Italy) to attend a comprehensive cardiac and nutritional rehabilitation program (duration 25 ± 3 days).

Inclusion criteria for participating in the study were (1) being 18 or over; (2) presenting a diagnosis of IHD, defined as a history of at least one of the following: myocardial infarction, coronary artery bypass grafting, or percutaneous transluminal coronary angioplasty; (3) presenting a history of congestive heart failure (CHF) with either reduced or preserved EF; (4) having BMI ≥ 30; and (5) having undergone symptom-limited exercise stress testing to define their effort tolerance for exercise.

Patients (1) with recent (less than 2 months) myocardial infarction, coronary artery bypass, or coronary angioplasty or (2) who were unable to perform ET were excluded from the study.

All patients were clinically stable, and none had clinically evident heart failure according to the Framingham criteria (McKee et al., 1971).

The study conformed with the principles outlined in the Declaration of Helsinki and complied with APA ethical standards.

### Measures

At the time of inclusion for CR, all subjects underwent a comprehensive routine assessment, including clinical history, physical examination, laboratory tests, and echocardiogram for the calculation of LVEF–which is the percentage of the diastolic left ventricular volume that is pumped out during systole–and exercise stress test. Also, demographic (i.e., age, gender, education) and behavioral (smoking status) parameters were collected at baseline.

### Exercise Test Protocol

A Marquette series 2000 motorized treadmill and Marquette Max Personal electrocardiography (ECG) instrumentation (Marquette Medical Systems, Milwaukee, WI, United States) were used to assess patients’ EC for treadmill speed and grade. EC is defined as the maximal oxygen intake for a stated workload, and it is commonly measured in metabolic equivalents (METs).

The test was tailored to patients’ characteristics, and a ramp protocol was used. ET was conducted in the morning, at least 2 h after breakfast and while taking regular medication.

Patients were encouraged to continue exercising until symptoms prevented them from continuing, even after reaching 85% of their maximum predicted heart rate. Reasons for test termination were limiting symptoms (i.e., fatigue, angina, dyspnea, and muscular pain), abnormal ECG, abnormal blood pressure, or choice of the patient.

Since cardiorespiratory parameters defined in normal weight populations differ from those of patients with obesity, the following *ad hoc* validated formula that takes into account BMI along with other variables (i.e., height, age, and gender) was used to predict EC: Predicted *EC (METs)* = *14.53 − (0.12 × age in years) −(0.17 × BM in kg/m^2^I)* + *(3.16 × height in meters) (*+ *0.71 for male patients)* (Gondoni et al., 2006). The ratio between measured and predicted values was calculated.

### Psychological Assessment

The Italian version of the *psychological general well-being index (PGWBI)* was used to investigate the patients’ self-evaluation of their perceived PWB. The score is derived from the summary score of six dimensions through 22 items: anxiety, depressed mood, positive well-being, self-control, general health, and vitality (Grossi et al., 2006). The PGWBI has been translated and culturally adapted into several languages across diverse populations of chronic patients and displays high values of Cronbach’s alpha coefficients (range 0.80–0.94). The majority of applications have been in studies involving patients with CVD (Croog et al., 1986). The overall reliability index for the present sample was 0.87. Data were obtained retrospectively from the patients’ medical records by a research assistant.

For the aims of the present study, literature-based selected predictors of EC were perceived PWB, education, LVEF, DM, AF, and smoking status.

Since BMI, height, age, and gender are parameters already included in the equation used to predict EC, these variables were excluded from the analysis.

### Statistical Analysis

Data were examined prior to hypothesis testing for identification of any missing data and normality, revealing no missing data that was normally distributed.

Descriptive statistics were performed to summarize the characteristics of the sample.

A multiple regression analysis was performed to evaluate whether DM, LVEF, AF, smoking status, education, and the PGWBI total score were significant and independent predictors of EC. The model was fitted by a forward stepwise method. Parametric assumptions, possible outliers, and influential cases were assessed by inspection of diagnostic plots and calculation of *ad hoc* statistics. Critical alpha was set at 0.05, if not otherwise specified, and *p*-values ≤ 0.05 were considered as statistically significant.

A Bayesian method for the evaluation of informative hypotheses on regression coefficients was also used to compare the predictors for statistical differences and identify the most influential ones (Kluytmans et al., 2012).

Descriptive statistics and regression analysis were completed with SPSS 20.0 for windows (Release 20.0.0, SPSS Inc.), while the BIEMS free software package (Mulder et al., 2009) was used to perform Bayesian analysis.

## Results

Of 1968 patients, 620 were female (31.5%) and 1348 were male (68.5%). The mean age of the sample was 62.2 (SD = 9.5), and the average BMI was 38.8 (SD = 5.4) (Table 1).

**TABLE 1:** Descriptive statistics of the sample.

	Total sample = 1968	
	*Mean (Min–Max)*	*SD*
Age (years)	62.2 (19.7–84.7)	9.5
Body mass index–BMI (kg/m^2^)*	38.8 (30–61.9)	5.4
Left ventricular eject fraction–LVEF (%)	0.6 (0.15–0.8)	0.1
Exercise capacity–EC (METs)	5.76 (2–16.8)	2.5
EC measured/predicted (METs)	93.03 (24–232.8)	32.5
Psychological general well-being–PGWBI total score	69.39 (6–136)	19.9
Gender (*n*;%)		
Male	1348	68.5
Female	620	31.5
Smoking status (*n*;%)		
Active	341	17.3
Past	1113	56.6
Never	517	26.1
Education** (*n*;%)		
Low	565	28.7
Middle	780	39.6
High	623	31.7
Coronary artery disease, CAD Ischemic heart disease, IHD (*n*;%)	1400	71.1
Chronic heart failure, CHF, with either reduced or preserved eject fraction, EF (*n*;%)	932	47.4
Type 2 diabetes–DM (*n*;%)	927	47.1
Atrial fibrillation–AF (*n*;%)	260	13.2

*Obesity levels: Class I obesity: 496 (25%), Class II obesity: 803 (41%), Class III obesity: 669 (34%). **Education was coded as follows: 5 years or less = low; 6 to 12 years = middle; >12 (i.e., high school or university) = high.

### Predictors of EC

The stepwise multiple regression analysis stopped at step 6 and the final model included LVEF, PGWBI, DM, smoking, AF, and education as significant and independent predictors of EC [*F*(6.1961) = 38.53; *p* < 0.001]. The multiple correlation coefficient was 0.32, indicating that approximately 10.5% of the variance of EC could be explained by the included variables.

Diagnostic statistics showed no collinearity among the predictors (VIF values were all well below 4 and the tolerance statistics all well above 0.2) (Hair et al., 2010), and the P–P plot, as well as the histogram, suggested normality of residuals. However, a relatively random display of points in the scatterplot of standardized residuals against standardized predicted values did not provide evidence of homogeneity of variance.

The Durbin-Watson statistic was also computed to evaluate independence of errors; the result was equal to 0.21, which was not considered acceptable.

Inspection of standardized residuals revealed that heteroskedasticity did not concern the LVEF and AF variables. With respect to outliers and influential cases, no more than 5% of cases were shown to have absolute standardized residuals above 2 (3.6%; *n* = 71). Still, 15 cases (0.76%) had standardized residuals equal to or above 3 and could thus be considered as outliers. However, neither the Mahalanobis and Cook distances, nor the DFBeta values, showed any case significantly influencing the estimation of the model parameters.

### Bayesian Analysis

Standardized regression coefficients (βs) showed LVEF to be the strongest predictor of EC in the study sample, followed by the PGWBI total score, DM, smoking status, and AF (Table 2).

**TABLE 2:** Summary of stepwise regression analyses for variables predicting exercise capacity (*n* = 1968).

	*B*	SE	β	*t*	Sig.
(Constant)	48.32	4.65		10.39	0.000
LVEF	62.80	6.25	0.21	10.05	0.000
PGWBI	0.23	0.35	0.14	6.58	0.000
Diabetes	–6.17	1.40	–0.95	–4.41	0.000
Smoking	–5.35	1.08	–0.11	–4.96	0.000
AF	–8.95	2.09	–0.09	–4.28	0.000
Education	2.53	0.91	0.06	2.79	0.005
*R*^2^	0.10				
*F*	38.53^∗^				

**^∗^p* < 0.01.*

However, this ranking is purely descriptive and provides no statistical evidence about the superiority of LVEF over PWB, or of PWB over the other determinants in predicting EC in the present population. Three informative hypotheses were thus formulated: (1) β_LVEF_ > β_PWB_, (2) β_PWB_ > β_DM_, and (3) β_PWB_ > β_Smoking_.

BIEMS generated a default prior and calculated a Bayes Factor for each hypothesis versus its unconstrained alternative (both βs are free to vary from minus to plus infinity).

For the first hypothesis, a Bayes factor of 1.96 was found. This means that the hypothesis stating that LVEF is more important for predicting EC than the PGWBI received almost two times more support from the data than the unconstrained hypothesis.

The second hypothesis received a Bayes factor of 2.00 and the third one received a value of 2.01, indicating that the data supported the superiority of PGWBI over both DM and smoking status in predicting EC.

## Discussion

This study targeted a large representative sample of patients with CVD and comorbid obesity referred for CR and revealed PWB to be a strong predictor of EC, second only to LVEF. Indeed, left ventricular dysfunction has been shown to have a largely demonstrated negative impact on exercise performance (von Roeder et al., 2017; Sacre et al., 2018), especially in obese patients (Benge et al., 1980). Moreover, DM, smoking status, AF, and education were significant predictors of EC in the present sample, although to a lesser extent than the subjective well-being.

Consistently, previous studies showed reduced EC in individuals without CVD who had DM (Choe et al., 2018), and a lower improvement of exercise tolerance in CR patients with diabetes compared with their non-diabetic counterparts–independently from their BMI (Verges et al., 2004; Kim et al., 2015).

These findings might be explained by the strong association between subjective well-being and lifestyle habits in people with DM. Diabetes is a chronic disease that affects both health and PWB (Moreno and Pearson, 2011). In fact, diet and exercise are the foundation of most therapies for diabetes (Tonetto et al., 2019), and people with DM often feel challenged by their disease-related features and complications and its day-to-day management demands (Thapa et al., 2019). Fears about the reality of complications (i.e., obesity, CVD, renal failure, etc.) also prevent people from exercising (Stryker, 2016) and are responsible for impaired PWB in persons with DM (Jing et al., 2018). At the same time, feeling good and staying healthy would give a further boost to the subjective well-being of the sufferers and their health-related outcomes. Accurate lifestyle analysis of patients suffering from CVD and other chronic conditions is, therefore, important for a comprehensive assessment in CR and the promotion of disease prevention strategies (Ceccarini et al., 2015; Pietrabissa et al., 2017b). This includes the screening of smoking habits and of socioeconomic status (SES) of the person.

In fact, cigarette smoking is a powerful independent risk factor for CVD and has a significant negative impact on EC (Asthana et al., 2012) and other cardiovascular parameters (Unverdorben et al., 2008). Still, most smokers gain weight after quitting due to increased energy intake and reduced energy expenditure. In order to prevent or reduce the onset of further complications, such as diabetes or obesity, actions for smoking cessation should, therefore, be combined with weight control interventions. These must be tailored for home-based care that would consider social disparities and meet the varying needs (and limitations) of the patients (Favoccia et al., 2014).

In fact, previous research has found inverse relationships between SES–including household income, education, housing status, and occupation–and unhealthy behaviors (Lantz et al., 2001; Pampel et al., 2010). In fact, socioeconomically challenged individuals confront more barriers (e.g., access, cost) to modifying risk behaviors over time such as quitting smoking, improving diet, increasing PA, and adhering to medications (Chandrasekhar, 2019). In particular, low education levels might affect the individuals’ ability to interpret and to comply with health recommendations and to interface effectively with healthcare professionals (Kavanagh et al., 2010; Stringhini et al., 2010; Prag and Subramanian, 2017; Andrade and Mehta, 2018; Vonneilich et al., 2019), therefore constituting a risk factor for adherence to PA programs and functional EC (Anderson et al., 1997; Shishehbor et al., 2006).

### Strengths and Limitation of the Study

The strengths of this study include its large sample size and the use of the Bayesian approach, which provides the most theoretically defensible framework that can be used to address probabilistic questions.

However, this work must be considered in light of several potential limitations. Firstly, absence of a comprehensive screening of mental health problems [e.g., eating disorders (Faulconbridge et al., 2012) and depression (Alizai et al., 2015; González-Castro et al., 2019)], readiness to change, and perceived self-efficacy of the participants prevented us from clarifying the role played by emotional factors in influencing the amount of physical effort that an individual with obesity and cardiac complication can sustain. Adaptation to living with CVD may, in fact, differ for each individual, and further studies should also investigate the role played by disease severity/typology (i.e., coronary artery disease–CAD, ischemic heart disease–IHD, or chronic heart failure–CHF) in the relation between PWB and patient-reported EC. Moreover, obesity might affect CR in many ways, but the absence of a control condition did not allow us to investigate the specific impact of PWB on EC during CR in both obese and non-obese people with CVD. It is also possible that other unmeasured modifiable and non-modifiable variables might account for these results, including family history, blood pressure, pulmonary functions, the skeletal muscle status, or the individuals’ perceived social support. Lastly, the use of self-report methods in the assessment of the individuals’ PWB and the absence of a proper cognitive screening in CR might limit the reliability of the research findings. Socially desirable responding, difficulties in assessing themselves accurately or misunderstanding of the meaning of the questions cannot, therefore, be excluded. Still, self-reports represent an inexpensive tool that allows the collection of a large amount of data in a relatively short amount of time and, therefore, are a particularly useful approach in assisting the assessment of CR patients in research and in everyday practice.

## Conclusion

The present study showed for the first time the independent impact of PWB on exercise intolerance over and above a series of selected physical and social components in a representative sample of patients with obesity and CVD enrolled in a CR program.

Exercise capacity is a key outcome of CR, and these results highlight the importance for healthcare professionals to properly assess the psychosocial status of the patient in order to promptly address potentially modifiable barriers to PA other than the biomedical ones (Baldasseroni et al., 2016; Cattivelli et al., 2018). Indeed, many patients with obesity and CVD fail to meet recommended daily PA levels (Balady and McInnis, 1996; Ghashghaei et al., 2012), and effective personalized actions aimed at increasing long-term behavioral change should be promoted (Pietrabissa et al., 2013, 2015, 2017a,b; Ceccarini et al., 2015).

Maintenance of healthy behaviors (i.e., PA, smoking, alcohol use, diet) would also increase cardiovascular parameters and reduce severity of comorbid illnesses (i.e., obesity, diabetes, etc.), thus preventing these conditions from further affecting the individuals’ exercise tolerance.

Still, longitudinal studies are needed to examine the mediating effect of health behaviors in the relationship between emotional status and the main clinical parameters of cardiac patients–with and without obesity. Findings from this study should be further explored in investigations targeting different populations. In fact, a research gap still exists in understanding the predictors and barriers to PA adherence in samples with varying background, ethnicity, or SES.

## Data Availability Statement

The datasets generated for this study are available on request to the corresponding author.

## Ethics Statement

The studies involving human participants were reviewed and approved by the Istituto Auxologico Italiano, IRCCS. The patients/participants provided their written informed consent to participate in this study.

## Author Contributions

LG conceived the original idea. GP planned and supervised the work, collected the experimental data, and wrote the manuscript with support from GC and RC. GM performed the analysis. EM supervised the findings of this work. All authors discussed the results and contributed to the final manuscript.

## Conflict of Interest

The authors declare that the research was conducted in the absence of any commercial or financial relationships that could be construed as a potential conflict of interest. The handling Editor declared a shared affiliation, though no other collaboration, with several of the authors GP, GC, RC, EM at the time of the review.
